# Bioactivity Evaluation of Plant Extracts Used in Indigenous Medicine against the Snail, *Biomphalaria glabrata*, and the Larvae of *Aedes aegypti*

**DOI:** 10.1155/2012/846583

**Published:** 2011-12-11

**Authors:** Edilson Alves dos Santos, Cenira M. de Carvalho, Ana L. S. Costa, Adilva S. Conceição, Flávia de B. Prado Moura, Antônio Euzébio Goulart Santana

**Affiliations:** ^1^Laboratório de Produtos Naturais, Instituto de Química e Biotecnologia, Universidade Federal de Alagoas, 57072-970 Maceió, AL, Brazil; ^2^Universidade do Estado da Bahia, Herbário da Universidade do Estado da Bahia, HUNEB-Coleção, 48608-230 Paulo Afonso, BA, Brazil; ^3^Departamento de Educação, Universidade do Estado da Bahia, Campus VIII, Rua do Bom Conselho 179, 48600-000 Paulo Afonso, BA, Brazil; ^4^Instituto de Ciências Biológicas e da Saúde, MHN, Universidade Federal de Alagoas, 57072-970 Maceió, AL, Brazil

## Abstract

This investigation examined the molluscicidal and larvicidal activity of eight plants that are used in the traditional medicine of the Pankararé indigenous people in the Raso da Catarina region, Bahia state, Brazil. The tested plants were chosen based on the results of previous studies. Only those plants that were used either as insect repellents or to treat intestinal parasitic infections were included in the study. Crude extracts (CEs) of these plants were tested for their larvicidal activity (against *Aedes aegypti* larvae in the fourth instar) and molluscicidal activity (against the snail *Biomphalaria glabrata*). The plant species *Scoparia dulcis* and *Helicteres velutina* exhibited the best larvicidal activities (LC_50_ 83.426 mg/L and LC_50_ 138.896 mg/L, resp.), and *Poincianella pyramidalis*, *Chenopodium ambrosoides*, and *Mimosa tenuiflora* presented the best molluscicidal activities (LC_50_ 0.94 mg/L, LC_50_ 13.51 mg/L, and LC_50_ 20.22 mg/L, resp.). As we used crude extracts as the tested materials, further study is warranted to isolate and purify the most active compounds.

## 1. Introduction

The Brazilian northeast is the poorest region of Brazil and has the worst Human Development Indices [1]. Most of this population is subjected to neglected tropical diseases that predominantly affect the poorest and most vulnerable groups, contributing to the perpetuation of poverty, inequality, and social exclusion [2].

Schistosomiasis and dengue fever cause major public health concerns in Brazil and other tropical developing countries. Schistosomiasis is caused by the parasite, *Schistosoma mansoni*, which uses the *Biomphalaria glabrata* snail as an essential intermediate host in its life cycle. Dengue fever is caused by an arbovirus of the Flaviviridae family and is transmitted by the mosquito, *Aedes aegypti*.

The number of cases of dengue has grown in Brazil, with epidemics in the most densely populated urban areas. However, natural products with different biocidal activities can help to fight parasite vectors at the adult or larval stages and can act as alternatives to synthetic products due to their rapid biodegradation and lower cost [3].

Molluscicides have been used as a general strategy to eliminate the snail that transmits schistosomiasis [4]. According to the World Health Organization (WHO), the use of drug therapy in conjunction with the use of molluscicides is the use the most valuable method to control schistosomiasis in areas with intermediary hosts. The synthetic substance, niclosamide (Bayluscide), has been used as the standard molluscicide since the 1960s, as it is efficient in controlling snails; however, the high cost of niclosamide and the fact that it decomposes rapidly in the presence of sunlight have limited the use of this drug [5].

Popular knowledge has been an important source of information for scientific research in several areas of study. Ethnopharmacological and ethnobotanical investigations have been used as the main strategy for selecting medicinal plants, thereby shortening the time for the discovery of new drugs, whereas ethnodirected research consists of selecting species based on information from population groups [6].

Evidence for the efficacy and safety and the immediate availability of plant-derived products for the control or eradication of such diseases would be of great value because part of the population living in the affected areas use plants and animals as one of the few options for disease treatment [7–9].

Studies have found evidence that standard methods control the dengue-related mosquito larvae with low efficacy, a situation that demonstrates the need for other means to fight the proliferation of dengue [10] given the fact that results in epidemiology are context-dependent [11]. Similarly, despite the fact that a national schistosomiasis control program was implemented in 1975, the disease still occurs in 19 states and is endemic to eight states.

Ethnobiological studies have been carried out on the indigenous Pankararé people since 1993 [12]. In 2006 [13], the use of 64 plants was reported, 20 of which were used for medicinal purposes. Indeed, there is evidence that the Pankararé—in the Estação Ecologica Raso da Catarina (a conservation area), Bahia state—have a profound knowledge regarding the benefits of plants.

This study examines the molluscicidal and larvicidal effects of eight plants used by the Pankararé indigenous people for medicinal purposes. The aim of the study is to look for evidence of alternative methods to fight vectors of schistosomiasis and dengue, taking into account local potentialities.

## 2. Materials and Methods

### 2.1. Study Area and Population

The indigenous lands of the Pankararé are located in one of the driest of the Brazilian regions, with an average annual rainfall of between 450 and 600 mm [14] and an average annual temperature of 25°C; the climate is arid and semiarid. The natural vegetation is tropical dry forest of the type hyperxerophylous steppic savanna.

The Pankararé have a long history of interaction with their regional neighbors and are a peasant social group that sees itself as a distinct ethnic group among the regional populations (from the social organization standpoint, this is termed Indigenous Peasantry). In Brazilian indigenous communities, the central political figure is the *Cacique* [15]. The Pankararé comprise a very poor group that has a long history of territorial disputes. They practice subsistence agriculture, farm livestock on a small scale, and engage in other activities, such as hunting, the collection of honey and wild fruits, and handicrafts [16].

### 2.2. Ethnobotanical Survey and Plant Collection

Ethnobiologic studies have been carried out on the Pankararé indigenous people since 1993. However, the present study was based mainly on recent information [17], specifically, on studies that were conducted in 2008 and 2009. The ethnobotanical research was carried out after the community members were fully informed of its purpose and they had granted their permission to record information. Authorization was also obtained from the community *Cacique* and the National Foundation of Support to the Indians (FUNAI). The data were collected using some of the methods presented and discussed in a previous report [18], including the use of informal conversations and semistructured interviews with 35 residents of the indigenous community (9 men and 26 women, aged 23 to 64 years). The interviewees were identified using the snowball technique based on the information initially provided by the traditional specialists, that is, people that are knowledgeable about medicinal plants.

For the taxonomic identification of the species, we collected reproductive plants that were donated to and identified in the Herbarium of the University of Bahia State UNEB (HUNEB-Coleção, Paulo Afonso).

### 2.3. Obtaining the Extracts

Eight plants were tested in bioassays to evaluate their larvicidal and molluscicidal activities. Plants that are used as insect repellents or anthelmintics were included in the study. To obtain the extracts, botanical material was gathered between March 2008 and February 2009. Material of the collected species (roots, stems, stem bark, and leaves) was dried at room temperature and then ground to powder. From this powder, we obtained the crude extract (CE) of each material by extracting three consecutive times with 90% ethanol for 72 h in a stainless steel extractor at 27 ± 1°C followed by the remotion of the solvent in a rotary evaporator at 60°C. The concentrated and weighed crude extracts were bioassayed to evaluate their larvicidal and molluscicidal activities at the Institute of Chemistry and Biotechnology (IQB) at the Federal University of Alagoas, UFAL.

### 2.4. Evaluation of the Molluscicidal Activity

Individual snails of the species *Biomphalaria glabrata* (of diameter 13–18 mm and obtained from the Institute René Rachou in Belo Horizonte city) were housed in aquaria equipped with continuously circulating dechlorinated water, at the temperature of 28°C, in the bioassay laboratory of the IQB of UFAL, based on the protocol of Santos and Sant'ana [19]. The ethanolic extracts of the stems, stem bark, roots, and leaves of the plants were dissolved in an aqueous solution of Dimethyl Sulfoxide (DMSO 0.1%) to obtain various concentrations (0.10–100 *μ*g/mL). The assay consisted of immersing five snails in 125 mL of the dissolved test extract for 24 hours. After this period, the snails were washed, placed in dechlorinated water, and fed. The snails remained under observation for a further 24 hours, and the dead snails were recorded and removed. The extracts that exhibited molluscicidal activity at concentrations lower than 100 *μ*g/mL in the duplicate bioassays were considered active and subjected to further tests. A total of 10 snails was used in these tests (maintaining the ratio of 25 mL of test solution per snail), and each concentration was tested in triplicate. In parallel, we carried out control tests using an aqueous solution of 0.1% DMSO with the molluscicide, niclosamide, at 3 *μ*g/mL. We used the program Probit, version 1.5 [55] to calculate the LC_50_.

The World Health Organization (WHO) has indicated that the crude extracts of plants presenting LC_50_ values of <40 ppm (0.04% and 0.4 *μ*g/mL) have potential as larvicidal and molluscicidal compounds [56].

### 2.5. Evaluation of the Larvicidal Activity

The larvicidal activity was tested on the larvae of the mosquito *Aedes aegypti* in the bioassay laboratory of the IQB/UFAL, based on methodology described by the WHO [57]. The crude extracts were diluted in aqueous solutions of 1% DMSO to 500 *μ*g/mL. Then, 10 larvae of *A. aegypti* in the fourth instar stage were prepared for immersion in the crude extract (20 mL). The larvae were counted 24 hours and 48 hours from the beginning of the experiment, and the tests were performed in quadruplicate. For the control, we used an aqueous solution of 1% DMSO containing 3 *μ*g/mL of the synthetic larvicide, Temephos. The activity of the tested extracts was established based on the average percentage of mortality of the larvae after 48 hours (>75% [promising result], 50–75% [partially promising], 25–50% [weakly promising], and <25% [inactive]).

### 2.6. Data Analysis

The results are reported as the average of three repetitions (*n* = 3) ± the standard deviation of the mean. The values found were subjected to ANOVA and Tukey's test (*P* ≤ 0.05). All of the analyses were performed using the Microcal Origin, version 8.0, and GraphPad Prism, version 5.0, programs.

## 3. Results and Discussion

The eight plant species examined belong to six families and eight genera, and a biological activity has previously been described in the literature for these species (Table 1). These species were cited by the Pankararé as insect repellents (5 species) and/or anthelmintics (4 species). The analyses of the extracts and the root, stem, and leaf fractions demonstrated that seven (87.5%) of the eight tested species presented activity. Five species (62.5%) were active against the mollusk, *Biomphalaria glabrata*, and two species (25%) were active against larvae of the *Aedes aegypti* mosquito.

Two of the seven species with activity (*Chenopodium ambrosioides* L. and *Hyptis pectinata *Poit.) are cultivated in home gardens. The remaining five species (*Helicteres velutina *K. Schum.,* Jatropha mollissima *(Pohl) Baill.,* Poincianella pyramidalis* (Tul.) L.P. Queiroz,* Mimosa tenuiflora *(Willd.) Poir., and* Scoparia dulcis *L.) are native or ruderal species.

The species with the strongest activity against the larvae of *A. aegypti* was *S. dulcis*, whose ethanolic leaf extract presented an LC_50_ 83.426 mg/L. The next strongest activity was presented by *Helicteres velutina *K. Schum., with an LC_50_ of 138.89 mg/mL and 171.68 mg/L, respectively, for the extracts obtained from the stems (215 g) and roots (208 g) (Table 2).

**Figure 1:**
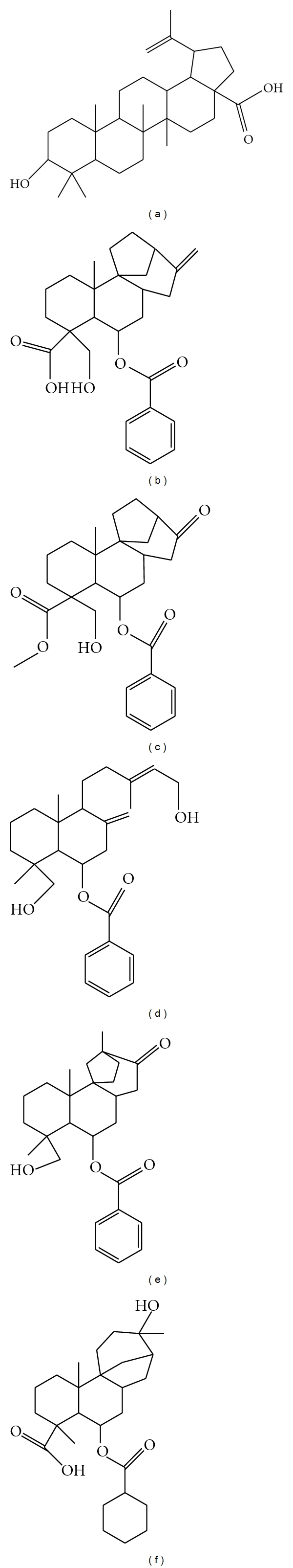
Chemical structures of selected compounds isolated from *S. dulcis*: (a) betulinic acid, (b) scopadulcic acid A, (c) scopadulcic acid B, (d) scopadiol, (e) scopadulciol, and (f) scopadulin.

Previous studies report that the extracts from the roots and stems of *S. dulcis* present a high activity against the bacterium *Klebsiella pneumoniae *and gram-negative bacteria [45]. Such results may be associated with the presence of steroids, saponins, polyphenols, and glutinol [58]. Flavonoids were also described as occurring in the aerial parts of *S. dulcis* [59], which suggests the presence of antioxidant activity. *S. dulcis* is a good source of betulinic acid (Figure 1), a compound with proven anticarcinogenic, antimelanoma, antiviral, and cytotoxic properties. Many biological activities that were found in *S. dulcis *have been attributed to various compounds, including scopadulcic acids A and B, scopadiol, scopadulciol, and scopadulin [60] (Figure 1).

No data were found in the literature that could be used in a comparative analysis of *Helicteres velutina*, a species whose roots and stems exhibited activity in the present study; such previously reported data were found only for *Helicteres angustifolia *L. [33]. The other species tested did not present activity against the larvae of *A. aegypti* using the applied method.

*P. pyramidalis* (Tul.) L.P. (LC_50_ 0.94 mg/L), *C. ambrosoides* (LC_50_ 13.51 mg/L), *M. tenuiflora* (LC_50_ 20.22 mg/L), *H. pectinata* (LC_50_ 25.55 mg/L), and *J. molissima* (LC_50_ 33.55 mg/L) demonstrated the strongest molluscicidal activities (Table 3). The extracts of *C. moritibensis *(the stems, roots, leaves, stem bark, and root bark) did not exhibit any significant activity, as based on the recommendations of the WHO [56]. These recommendations propose that only the aqueous and alcoholic extracts of plants that promote the death of 90% of the animals when tested at concentrations equal to or lower than 20 mg/L (over a 24-hour exposure) should be tested in the field and deserve attention in studies on the purification and isolation of the most active compounds.

**Figure 2:**
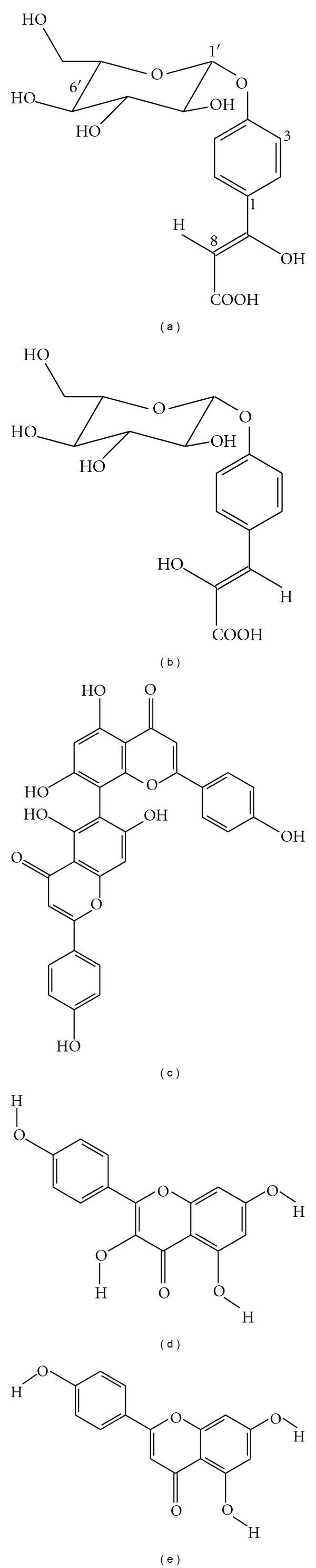
Chemical structures of some compounds isolated from* P. pyramidalis*: (a) apigenin, kaempferol and (b) agathisflavone.

The ethanolic extract of *P. pyramidalis* has been used successfully against resistant strains of *Escherichia coli* (strain ATCC25922) and *Staphylococcus aureus* (ATCC25923) [48]. A phytochemical study of this species revealed the presence of phenolic compounds, such as glucosyl-phenylpropanoid acid, 4-Obd-glucopyranosyloxi-Z-7-hydroxycinnamic acid and 4-O-b-glucopyranosyloxi-Z-8-hydroxycinnamic acid, and flavonoids agathisflavone, apigenin, and kaempferol [53] (Figure 2).

The excellent efficacy of this species is important, and the crude extract deserves further studies with regard to the purification and isolation of the most active compounds. Importantly, *P. pyramidalis* occurs frequently and this species grows densely in semiarid dry forest in northeastern Brazil, locally known as caatinga [61], presenting the strong potential for large-scale exploitation without incurring a major risk to its population and the possibility of generating income for local communities and creating major public health benefits.

Mastruz (*C. ambrosoides*), which was the second most efficacious species, provides an essential oil containing the active ingredient monoterpene ascaridol [62]. The essential oil of this species has been used as an anthelmintic in humans but, due to its high toxicity, it has been replaced with safer drugs [63].

In a review on the phytochemistry of jurema-preta, Souza et al. [64] report the presence of several metabolites, such as alkaloids, chalcones, steroids, terpenoids, and phenoxychromones. Choi et al. [65] has suggested that many biological plant activities are due to the presence of total phenols, such as flavonoids and tannins.

Sambacaitá (*H. pectinata*), which is also bioactive, has proven anti-inflammatory, hepatoprotective, antibacterial, antifungal, and antinociceptive action [49–54].

The bark extract from *J. mollissima* was more toxic to the* A. aegypti* larvae than the leaf or root extracts. These results are similar to those found by Heal et al. [66].

**Figure 3:**
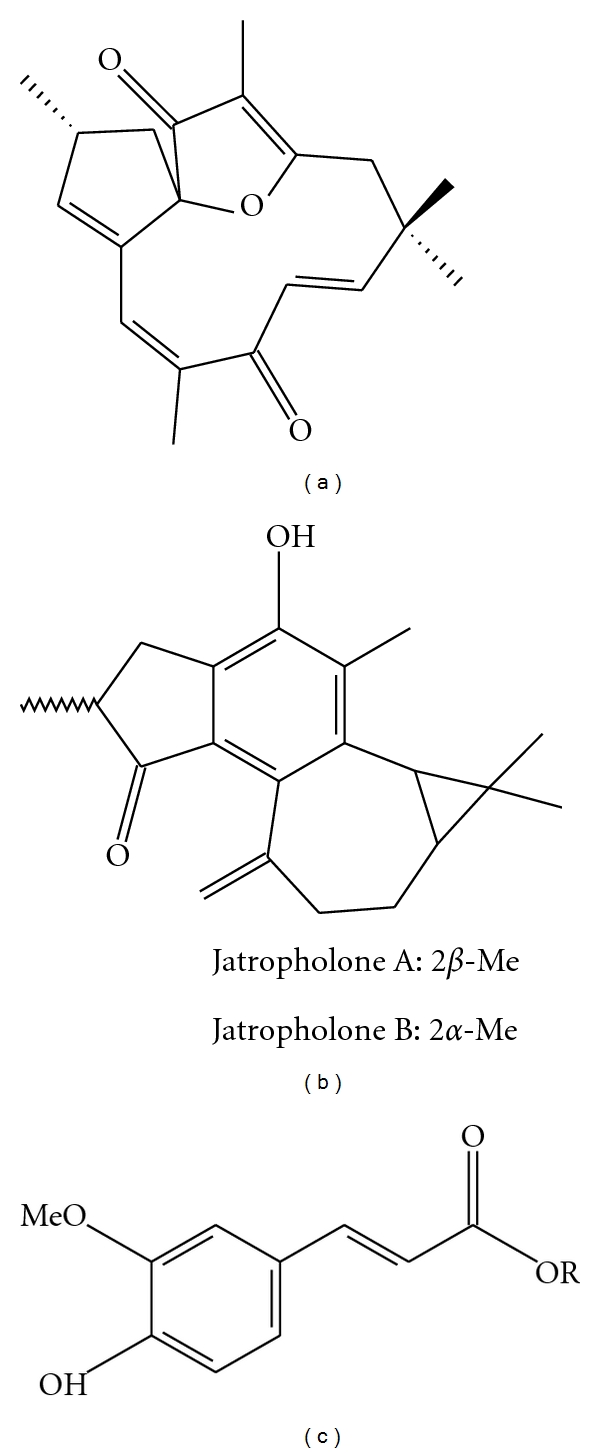
Chemical structures of some compounds isolated from plants of the genus *Jatropha*: (a) jatrophone, (b) jatropholone A and B, (c) ferulates.

The diterpenoids, jatrophone, and jatropholones A and B (Figure 3), are classified as cytotoxic substances, and the isolation of tumor growth inhibitors from this plant has been cited by Goulart et al. [67] and Kupchan et al. [68, 69]. These substances have been the subject of several scientific studies and have been reported to inhibit the effects of insulin [70]. 

Although previous researchers have studied the bioactive substances of seven of the eight species examined here, no reference was found concerning its molluscicidal or larvicidal potential.

Although there is a need for further investigation to isolate and identify the constituents of the crude plant extracts studied, the results of this study provide evidence that some of the plants tested contain natural insecticides and have the potential for larvicidal activity and may provide substitutes for synthetic products in the future. Developments in this direction may lead to important positive impacts on public health programs, particularly with regard to socially excluded populations.

## 4. Conclusion

During the molluscicidal tests, we verified that the leaf extracts of *Poincianella pyramidalis* and *Chenopodium ambrosoides* were very effective and had a high level of activity. However, other extracts were more promising for larvicidal activity; of particular note were the leaf extracts from *Scoparia dulcis* and the stem and root extracts of *Helicteres velutina*. Based on these results, the plants of the studied region have proven to be a rich source of substances with larvicidal activity (based on *Aedes aegypti* larvae in the fourth instar) and molluscicidal activity (based on the snail *Biomphalaria glabrata*). Further attention is warranted for the purification and isolation of the most active compounds from these extracts, and field tests are needed. From the information presented here, the need for an interdisciplinary research program in the northeastern semiarid region is evident, especially in the village of the Pankararé people. Such a program would be aimed at developing the sustainable use of natural resources and promoting community development.

## Figures and Tables

**Table 1:** Chemical composition and pharmacological activities of the plants used by the Pankararés, Bahia, northeast Brazil, as repellents or anthelmintics.

Popular name	Family/Species	Part used	Biological activity	Isolated substances	References
Pinhão branco^B^	euphorbiaceae/ *Jatropha mollissima *(Pohl) Baill.	Leaves, stem bark, and roots	Antioxidant	*	[20]

Jurema preta^B^	fabaceae/ *Mimosa tenuiflora* (Willd.) Poir.	Leaves, stem bark, and roots	*	Kukulkanins A and B	[21]

Velame	euphorbiaceae/ *Croton moritibensis* Baill.	Leaves and aerial parts	*	12-hydroxy-13-methyl-1,8,11,13-podocarpatetraen-3-one, 2-ethoxycarbonyltetrahydroharman and hydroxy-2-methyltetrahydroharman	[22]

Vassourinha^B^	plantaginaceae/ *Scoparia dulcis* L.	Aerial parts	Antidiabetic, antioxidant, cytotoxic, antiulcer, antileishmania, and anti-hyperlipidemic	Scoparic acid D	[23–32]
Pitó^A^	sterculiaceae/ *Helicteres velutina *K. Schum.	Aerial parts	Cytotoxic against various cancer lineages**	Cucurbitacins D and J**	[33]
Mastruz^B^	chenopodiaceae/ *Chenopodium ambrosioides* L.	Aerial parts	Inhibitor of the mitochondrial electron transport chain (toxicity), antiparasitic, antihyperperistalsis, anti-inflammatory, analgesic, antischistosomiasis, antituberculosis, larvicidal, antitumor, and antifungal	Caryophyllene oxide	[34–44].
Catingueira^A,B^	fabaceae/ *Poincianella pyramidalis *(Tul.) L.P. Queiroz	Leaves, stem bark and roots	Antimicrobial, antioxidant and antifungal	4,4′-dihydroxy-2′-methoxy-chalcone	[45–48].
Sambacaitá^B^	lamiacae/ *Hyptis pectinata* L.	Aerial parts	Antimicrobial, antinociceptive, anti-inflammatory, hepatoprotective, antibacterial, and antifungal	Calamusenone-antimicrobial	[49–54].

*No studies were found in the literature; **activity has been described for another species of the same genus; ^A^larvicidal activity; ^B^molluscicidal activity.

**Table 2:** In vitro larvicidal activity of ethanolic extracts from *Scoparia dulcis* and *Helicteres velutina*. LC: Lethal concentration reported as LC_10_, LC_50_, and LC_90_ (in mg/L).

Tested species	Popular name	Part used	LC_10_	LC_50_	LC_90_
*Scoparia dulcis*	Vassourinha	Leaf	43.820	83.426	158.829
*Helicteresvelutina*	Pitó	Root	73. 029	171.683	403.607
*Helicteresvelutina*	Pitó	Stem	60.406	138.896	319.372

**Table 3:** Molluscicidal activity against *B. glabrata* of plant extracts used by the Pankararé indigenous people (Bahia State, Brazil). LC: Lethal concentration reported as LC_10_, LC_50_, and LC_90_ (in mg/L).

Crude extract	Common name	Used part	LC_10_	LC_50_	LC_90_
*Jatropha mollissima*	Pinhão-branco	Stem	20.00	33.55	56.26
*Hyptis pectinata*	Sambacaetá	Leaf	8.48	25.34	75.66
*Poincianella pyramidalis*	Catingueira	Leaf	0.04	0.94	20.03
*Chenopodium ambrosoides*	Mastruz	Leaf	1.99	13.51	91.57
*Mimosa tenuiflora*	Jurema preta	Stem	6.59	20.22	62.05
